# Mortality Risk Among Patients Hospitalized Primarily for COVID-19 During the Omicron and Delta Variant Pandemic Periods — United States, April 2020–June 2022

**DOI:** 10.15585/mmwr.mm7137a4

**Published:** 2022-09-16

**Authors:** Stacey Adjei, Kai Hong, Noelle-Angelique M. Molinari, Lara Bull-Otterson, Umed A. Ajani, Adi V. Gundlapalli, Aaron M. Harris, Joy Hsu, Sameer S. Kadri, Jon Starnes, Kristin Yeoman, Tegan K. Boehmer

**Affiliations:** ^1^CDC COVID-19 Emergency Response Team; ^2^Clinical Epidemiology Section, Critical Care Medicine Department, National Institutes of Health Clinical Center, Bethesda, Maryland; ^3^Booz Allen Hamilton, Inc., McLean, Virginia.

The risk for COVID-19–associated mortality increases with age, disability, and underlying medical conditions (*1*). Early in the emergence of the Omicron variant of SARS-CoV-2, the virus that causes COVID-19, mortality among hospitalized COVID-19 patients was lower than that during previous pandemic peaks (*2*–*5*), and some health authorities reported that a substantial proportion of COVID-19 hospitalizations were not primarily for COVID-19–related illness,* which might account for the lower mortality among hospitalized patients. Using a large hospital administrative database, CDC assessed in-hospital mortality risk overall and by demographic and clinical characteristics during the Delta (July–October 2021), early Omicron (January–March 2022), and later Omicron (April–June 2022) variant periods^†^ among patients hospitalized primarily for COVID-19. Model-estimated adjusted mortality risk differences (aMRDs) (measures of absolute risk) and adjusted mortality risk ratios (aMRRs) (measures of relative risk) for in-hospital death were calculated comparing the early and later Omicron periods with the Delta period. Crude mortality risk (cMR) (deaths per 100 patients hospitalized primarily for COVID-19) was lower during the early Omicron (13.1) and later Omicron (4.9) periods than during the Delta (15.1) period (p<0.001). Adjusted mortality risk was lower during the Omicron periods than during the Delta period for patients aged ≥18 years, males and females, all racial and ethnic groups, persons with and without disabilities, and those with one or more underlying medical conditions, as indicated by significant aMRDs and aMRRs (p<0.05). During the later Omicron period, 81.9% of in-hospital deaths occurred among adults aged ≥65 years and 73.4% occurred among persons with three or more underlying medical conditions. Vaccination, early treatment, and appropriate nonpharmaceutical interventions remain important public health priorities for preventing COVID-19 deaths, especially among persons most at risk.

COVID-19 hospitalizations and in-hospital deaths during April 2020–June 2022 were identified from 678 hospitals in the Premier Healthcare Database Special COVID-19 Release (PHD-SR).^§^ COVID-19 hospitalizations were defined as those with the *International Classification of Diseases, Tenth Revision, Clinical Modification* (ICD-10-CM) code U07.1 (COVID-19, virus identified [laboratory-confirmed]) listed as the primary or secondary discharge diagnosis; a COVID-19 in-hospital death was defined as a COVID-19 hospitalization with expired discharge status. COVID-19 hospitalizations were identified as being primarily for COVID-19 if they had 1) a U07.1 primary discharge diagnosis or 2) a U07.1 secondary discharge diagnosis accompanied by either treatment with remdesivir or a primary discharge diagnosis of sepsis, pulmonary embolism, acute respiratory failure, or pneumonia.^¶^ Monthly cMRs (deaths per 100 hospitalizations) were calculated for COVID-19 hospitalizations (total, primarily for COVID-19, and not primarily for COVID-19) and non–COVID-19 hospitalizations.

Patient-level analyses were conducted by selecting each patient’s last hospitalization primarily for COVID-19 during the Delta, early Omicron, and later Omicron periods. For each period, sociodemographic (age, sex, race and ethnicity, and insurance type), clinical (underlying medical conditions, disability status, and previous COVID-19),** disease severity (intensive care unit [ICU] admission, receipt of COVID-19 medications, noninvasive ventilation, and invasive mechanical ventilation [IMV]),^††^ and hospital (U.S. Census Bureau region and number of beds) characteristics were described for patients hospitalized primarily for COVID-19 and in-hospital deaths, and cMR was calculated. Descriptive analyses were also conducted for three pre-Delta periods (April–September 2020, October 2020–February 2021, and March–June 2021).

Using a generalized estimating equations model, specified as a log-linked binomial regression including all three periods, aMRDs and aMRRs for in-hospital death were estimated across periods (early Omicron versus Delta and later Omicron versus Delta).^§§^ aMRDs were estimated as the difference in the adjusted predicted mortality risk between periods; aMRRs were estimated as the ratio of adjusted predicted mortality risk between periods.^¶¶^ SEs and 95% CIs were obtained by hospital-patient clustered bootstrapping with 500 replications. Z-tests were used to compare cMR, aMRDs, and aMRRs among pandemic periods; p<0.05 was considered statistically significant. Analyses were conducted using SAS (version 9.4; SAS Institute) and Stata (version 15.1; StataCorp). This activity was reviewed by CDC and conducted consistent with applicable federal law and CDC policy.***

During April 2020–June 2022, a total of 1,072,106 COVID-19 hospitalizations and 128,517 in-hospital deaths were reported in PHD-SR. The proportion of COVID-19 hospitalizations identified as primarily for COVID-19 was relatively stable during the pre-Omicron period (83.8%, 95% CI = 83.7–83.9) and decreased during the Omicron period (62.8%, 95% CI = 62.6–63.0) (Supplementary Figure, https://stacks.cdc.gov/view/cdc/121070). cMR was 1–2 percentage points higher for hospitalizations primarily for COVID-19 than for total COVID-19 hospitalizations through December 2021; the cMR difference increased to 3–3.5 percentage points during the early Omicron period, when the proportion of hospitalizations primarily for COVID-19 and cMRs began decreasing, and returned to 1–2 percentage points in the later Omicron period (Figure).

**FIGURE:**
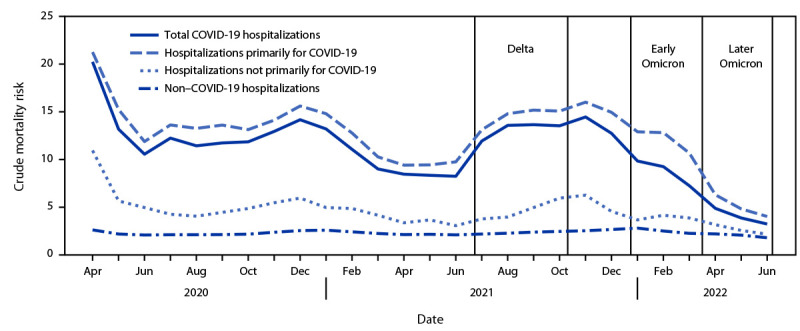
Crude mortality risk* for total COVID-19 hospitalizations, hospitalizations primarily for COVID-19, hospitalizations not primarily for COVID-19,^†^ and non–COVID-19 hospitalizations — Premier Healthcare Database Special COVID-19 Release,^§^ United States, April 2020–June 2022^¶^ * In-hospital mortality was defined by a discharge status of expired. Crude mortality risk was calculated as in-hospital deaths per 100 hospitalizations. ^†^ Total COVID-19 hospitalizations are those with a primary or secondary discharge diagnosis of COVID-19 (i.e., *International Classification of Diseases, Tenth Revision, Clinical Modification* code of U07.1). Non–COVID-19 hospitalizations are those without a COVID-19 discharge diagnosis. Hospitalizations primarily for COVID-19 had a primary discharge diagnosis of COVID-19 or a secondary discharge diagnosis of COVID-19 accompanied by either treatment with remdesivir or a primary discharge diagnosis of sepsis, pulmonary embolism, acute respiratory failure, or pneumonia. Hospitalizations not primarily for COVID-19 are those that did not meet criteria for a hospitalization primarily for COVID-19. ^§^ August 2, 2022, data release. Data are from 678 hospitals that had at least one inpatient record per month during April 2020–May 2022. ^¶^ Variant pandemic periods were selected based on two factors: 1) the U.S. epidemic curve for new admissions of patients with confirmed COVID-19 (https://covid.cdc.gov/covid-data-tracker/#new-hospital-admissions) and 2) the U.S. variant proportions from SARS-CoV-2 genomic surveillance (https://data.cdc.gov/Laboratory-Surveillance/SARS-CoV-2-Variant-Proportions/jr58-6ysp). Pandemic periods are defined using whole months because of date aggregation in the data source. The Delta variant (B.1.617.2) became the predominant circulating strain (representing >50% of sequenced isolates) during the week ending June 26, 2021, the Omicron B.1.1.529 subvariant became the predominant circulating strain during the week ending December 25, 2021, and the Omicron BA.2 subvariant became the predominant circulating strain during the week ending March 26, 2022. The predominant circulating strains during the early Omicron period were B.1.1.529 and BA.1 and during the later Omicron period were BA.2 and BA.2.12.1.

Among patients hospitalized primarily for COVID-19 who died in-hospital during the Delta, early Omicron, and later Omicron periods, 57.8%, 58.0%, and 51.4%, respectively, were male; 63.8%, 66.8%, and 69.1%, respectively, were non-Hispanic White (White); 53.7%, 73.5%, and 81.9%, respectively, were aged ≥65 years; 15.1%, 22.9%, and 28.9%, respectively, had a disability; and 61.7%, 70.8%, and 73.4%, respectively, had three or more underlying medical conditions (Table 1). In addition, a decreasing proportion of patients who died in-hospital had other indicators of disease severity during the Delta, early Omicron, and later Omicron periods: 76.1%, 64.0%, and 57.2%, respectively, were admitted to ICU; 93.8%, 86.8%, and 76.4%, respectively, received COVID-19 medications; 61.8%, 51.2%, and 35.0%, respectively, received noninvasive ventilation; and 71.9%, 57.6%, and 43.6%, respectively, received IMV.

**TABLE 1:** Characteristics of patients hospitalized primarily for COVID-19* and in-hospital deaths among patients hospitalized primarily for COVID-19^†^ during the Delta, early Omicron, and later Omicron pandemic periods^§^ — Premier Healthcare Database Special COVID-19 Release,^¶^ United States, July 2021–June 2022

Characteristic	No. (column %)					
	Delta		Early Omicron		Later Omicron	
	(Jul–Oct 2021)		(Jan–Mar 2022)		(Apr–Jun 2022)	
	Hospitalized patients	In-hospital deaths	Hospitalized patients	In-hospital deaths	Hospitalized patients	In-hospital deaths
**Total patients**	**163,094 (100)**	**24,658 (100)**	**104,395 (100)**	**13,701 (100)**	**20,655 (100)**	**1,004 (100)**
**Age group, yrs**						
0–17	2,219 (1.4)	15 (0.1)	2,073 (2.0)	10 (0.1)	690 (3.3)	6 (0.6)
18–34	14,187 (8.7)	683 (2.8)	4,230 (4.1)	167 (1.2)	875 (4.2)	8 (0.8)
35–49	32,353 (19.8)	3,017 (12.2)	9,453 (9.1)	610 (4.5)	1,415 (6.8)	29 (2.9)
50–64	51,208 (31.4)	7,696 (31.2)	26,258 (25.2)	2,842 (20.7)	3,691 (17.9)	139 (13.8)
65–79	43,707 (26.8)	9,044 (36.7)	38,648 (37.0)	5,896 (43.0)	7,063 (34.2)	371 (37.0)
≥80	19,420 (11.9)	4,203 (17.0)	23,733 (22.7)	4,176 (30.5)	6,921 (33.5)	451 (44.9)
**Sex**						
Male	85,553 (52.5)	14,241 (57.8)	54,153 (51.9)	7,951 (58.0)	9,978 (48.3)	516 (51.4)
Female	77,541 (47.5)	10,417 (42.2)	50,242 (48.1)	5,750 (42.0)	10,677 (51.7)	488 (48.6)
**Race and ethnicity**						
Hispanic or Latino	25,730 (15.8)	3,559 (14.4)	13,515 (12.9)	1,696 (12.4)	2,295 (11.1)	88 (8.8)
White, NH	100,601 (61.7)	15,733 (63.8)	67,786 (64.9)	9,151 (66.8)	13,961 (67.6)	694 (69.1)
Black or African American, NH	24,714 (15.2)	3,389 (13.7)	15,713 (15.1)	1,738 (12.7)	2,686 (13.0)	117 (11.7)
Asian, NH	2,575 (1.6)	380 (1.5)	2,098 (2.0)	307 (2.2)	634 (3.1)	34 (3.4)
Other, NH	6,544 (4.0)	1,071 (4.3)	3,673 (3.5)	555 (4.1)	703 (3.4)	46 (4.6)
Unknown	2,930 (1.8)	526 (2.1)	1,610 (1.5)	254 (1.9)	376 (3.8)	25 (2.5)
**Insurance type**						
Commercial	54,199 (33.2)	5,907 (24.0)	18,548 (17.8)	1,652 (12.1)	2,824 (13.7)	90 (9.0)
Medicare	67,361 (41.3)	13,705 (55.6)	65,874 (63.1)	10,152 (74.1)	14,382 (69.6)	798 (79.5)
Medicaid	23,521 (14.4)	2,722 (11.0)	13,810 (13.2)	1,195 (8.7)	2,446 (11.8)	77 (7.7)
Self-pay	5,966 (3.7)	754 (3.1)	1,780 (1.7)	196 (1.4)	329 (1.6)	9 (0.9)
Other/Unknown	12,047 (7.4)	1,570 (6.4)	4,383 (4.2)	506 (3.7)	674 (3.3)	30 (3.0)
No. of underlying medical conditions**						
0	25,191 (15.4)	704 (2.9)	7,844 (7.5)	246 (1.8)	1,451 (7.0)	9 (0.9)
1	39,060 (23.9)	3,171 (12.9)	16,117 (15.4)	1,262 (9.2)	3,015 (14.6)	87 (8.7)
2	36,200 (22.2)	5,561 (22.6)	20,869 (20.0)	2,494 (18.2)	3,967 (19.2)	171 (17.0)
3	26,944 (16.5)	6,021 (24.4)	20,665 (19.8)	3,149 (23.0)	4,097 (19.8)	216 (21.5)
4	17,416 (10.7)	4,451 (18.1)	16,681 (16.0)	2,809 (20.5)	3,482 (16.9)	216 (21.5)
≥5	18,283 (11.2)	4,750 (19.3)	22,219 (21.3)	3,741 (27.3)	4,643 (22.5)	305 (30.4)
**Disability^††^**						
Yes	18,654 (11.4)	3,712 (15.1)	21,176 (20.3)	3,144 (22.9)	5,131 (24.8)	290 (28.9)
No	144,440 (88.6)	20,946 (84.9)	83,219 (79.7)	10,557 (77.1)	15,524 (75.2)	714 (71.1)
**Previous COVID-19^§§^**						
Yes	580 (0.4)	53 (0.2)	1,797 (1.7)	123 (0.9)	860 (4.2)	28 (2.8)
No	162,514 (99.6)	24,605 (99.8)	102,598 (98.3)	13,578 (99.1)	19,795 (95.8)	976 (97.2)
**Intensive care unit admission**						
Yes	40,818 (25.0)	18,777 (76.1)	22,320 (21.4)	8,766 (64.0)	2,747 (13.3)	574 (57.2)
No	122,276 (75.0)	5,881 (23.9)	82,075 (78.6)	4,935 (36.0)	17,908 (86.7)	430 (42.8)
**Medication treatment** ^¶¶^						
Yes	148,328 (90.9)	23,117 (93.8)	84,459 (80.9)	11,892 (86.8)	14,857 (71.9)	767 (76.4)
No	14,766 (9.1)	1,541 (6.2)	19,936 (19.1)	1,809 (13.2)	5,798 (28.1)	237 (23.6)
**Noninvasive ventilation**						
Yes	35,680 (21.9)	15,247 (61.8)	18,829 (18.0)	7,013 (51.2)	2,167 (10.5)	351 (35.0)
No	127,414 (78.1)	9,411 (38.2)	85,566 (82.0)	6,688 (48.8)	18,488 (89.5)	653 (65.0)
**Invasive mechanical ventilation**						
Yes	28,367 (17.4)	17,739 (71.9)	14,049 (13.5)	7,894 (57.6)	1,260 (6.1)	438 (43.6)
No	134,727 (82.6)	6,919 (28.1)	90,346 (86.5)	5,807 (42.4)	19,395 (93.9)	566 (56.4)
**Hospital characteristics**						
U.S. Census Bureau region***						
Midwest	28,851 (17.7)	3,899 (15.8)	21,567 (20.7)	2,929 (21.4)	4,557 (22.1)	208 (20.7)
Northeast	10,350 (6.3)	1,361 (5.5)	14,090 (13.5)	1,850 (13.5)	4,542 (22.0)	243 (24.2)
South	96,857 (59.4)	15,203 (61.7)	51,701 (49.5)	6,581 (48.0)	8,652 (41.9)	393 (39.1)
West	27,036 (16.6)	4,195 (17.0)	17,037 (16.3)	2,341 (17.1)	2,904 (14.1)	160 (15.9)
**No. of hospital beds**						
0–199	43,939 (26.9)	5,559 (22.5)	25,537 (24.5)	2,747 (20.0)	4,731 (22.9)	183 (18.2)
200–499	75,271 (46.2)	11,932 (48.4)	49,725 (47.6)	6,892 (50.3)	9,467 (45.8)	478 (47.6)
≥500	43,884 (26.9)	7,167 (29.1)	29,133 (27.9)	4,062 (29.6)	6,457 (31.3)	343 (34.2)

**Abbreviation:** ICD-10-CM = *International Classification of Diseases, Tenth Revision, Clinical Modification*; NH = non-Hispanic.

* Patients hospitalized primarily for COVID-19 had a primary discharge diagnosis of COVID-19 (i.e., ICD-10-CM code of U07.1) or a secondary discharge diagnosis of COVID-19 accompanied by either treatment with remdesivir or a primary discharge diagnosis of sepsis, pulmonary embolism, acute respiratory failure, or pneumonia.

^†^ In-hospital deaths were patients with a discharge status of expired.

^§^ Variant pandemic periods were selected based on two factors: 1) the U.S. epidemic curve for new admissions of patients with confirmed COVID-19 (https://covid.cdc.gov/covid-data-tracker/#new-hospital-admissions) and 2) the U.S. variant proportions from SARS-CoV-2 genomic surveillance (https://data.cdc.gov/Laboratory-Surveillance/SARS-CoV-2-Variant-Proportions/jr58-6ysp). Pandemic periods are defined using whole months because of date aggregation in the data source. The Delta variant (B.1.617.2) became the predominant circulating strain (representing >50% of sequenced isolates) during the week ending June 26, 2021, the Omicron B.1.1.529 subvariant became the predominant circulating strain during the week ending December 25, 2021, and the Omicron BA.2 subvariant became the predominant circulating strain during the week ending March 26, 2022. The predominant circulating strains during the early Omicron period were B.1.1.529 and BA.1 and during the later Omicron period were BA.2 and BA.2.12.1.

^¶^ August 2, 2022, data release. Data are from 678 hospitals that had at least one inpatient record per month during April 2020–May 2022.

** Sixteen underlying medical conditions associated with higher risk for severe COVID-19 were assessed: asthma, cerebrovascular disease, cancer, chronic kidney disease, chronic lung disease, chronic liver disease, cystic fibrosis, dementia, diabetes, heart conditions, HIV, mental health disorder, obesity, primary immunodeficiencies, transplantation, and tuberculosis (https://www.cdc.gov/coronavirus/2019-ncov/science/science-briefs/underlying-evidence-table.html). Conditions were assessed using ICD-10-CM codes listed either at or before the COVID-19 health care encounter. For each patient, the number of underlying medical conditions was summed.

^††^ Presence of a disability was assessed using ICD-10-CM codes for birth defects, developmental disabilities, spinal cord injury, traumatic brain injury, and vision-, hearing-, and mobility-related disabilities.

^§§^ Previous COVID-19 was identified by presence of a COVID-19 diagnosis during an outpatient or inpatient encounter that occurred in the same hospital system ≥90 days before the current diagnosis.

^¶¶^ Patient treated with one of the following COVID-19 medications: dexamethasone, remdesivir, baricitinib, tofacitinib, tocilizumab, or sarilumab.

*** https://www2.census.gov/geo/pdfs/maps-data/maps/reference/us_regdiv.pdf

The cMR among patients hospitalized primarily for COVID-19 was 15.1 during the Delta, 13.1 during the early Omicron, and 4.9 during the later Omicron periods (Table 2); cMR range was 9.9–16.1 during the pre-Delta periods (Supplementary Table, https://stacks.cdc.gov/view/cdc/121069). After adjustment, in-hospital mortality was 0.69 (95% CI = 0.68–0.70) times as likely during the early Omicron period and 0.24 (95% CI = 0.22–0.25) times as likely during the later Omicron period than during the Delta period. Adjusted mortality risk during the early and later Omicron periods was lower than it was during the Delta period for patients aged ≥18 years, males and females, all racial and ethnic groups, persons with and without disabilities, and those with one or more underlying medical conditions, as indicated by significant aMRDs and aMRRs (p<0.05); mortality risk did not differ between the Omicron and Delta periods for patients aged <18 years. Larger aMRDs were observed with increasing age and number of underlying medical conditions; aMRD and aMRR were similar in magnitude for patients with and without disabilities.

**TABLE 2:** Crude mortality risk, adjusted mortality risk difference, and adjusted mortality risk ratio* among patients hospitalized primarily for COVID-19^†^ during the Delta, early Omicron, and later Omicron pandemic periods^§^ — Premier Healthcare Database Special COVID-19 Release,^¶^ United States, July 2021–June 2022

Characteristic	Crude mortality risk			Early Omicron versus Delta**		Later Omicron versus Delta**	
	Delta	Early Omicron	Later Omicron	Adjusted mortality risk difference (95% CI)	Adjusted mortality risk ratio (95% CI)	Adjusted mortality risk difference (95% CI)	Adjusted mortality risk ratio (95% CI)
	(Jul–Oct 2021)	(Jan–Mar 2022)	(Apr–Jun 2022)				
**Overall**	**15.1**	**13.1**	**4.9**	**−5.3 (−5.5 to −5.0)^††^**	**0.69 (0.68 to 0.70)^††^**	**−12.8 (−13.2 to −12.5)^††^**	**0.24 (0.22 to 0.25)^††^**
**Age group, yrs**							
0–17	0.7	0.5	0.9	−0.5 (−1.4 to 0.5)	0.64 (0.07 to 1.21)	0.5 (−1.3 to 2.4)	1.42 (−0.12 to 2.96)
18–34	4.8	3.9	0.9	−2.2 (−3.0 to −1.4)^††^	0.67 (0.56 to 0.78)^††^	−5.7 (−6.7 to −4.6)^††^	0.17 (0.03 to 0.31)^††^
35–49	9.3	6.5	2.0	−5.3 (−6.0 to −4.7)^††^	0.55 (0.51 to 0.60)^††^	−9.9 (−10.8 to −9.0)^††^	0.18 (0.11 to 0.24)^††^
50–64	15.0	10.8	3.8	−6.3 (−6.8 to −5.7)^††^	0.62 (0.60 to 0.65)^††^	−13.1 (−13.7 to −12.4)^††^	0.21 (0.18 to 0.24)^††^
65–79	20.7	15.3	5.3	−5.8 (−6.3 to −5.3)^††^	0.70 (0.68 to 0.72)^††^	−14.9 (−15.5 to −14.3)^††^	0.24 (0.21 to 0.26)^††^
≥80	21.6	17.6	6.5	−3.2 (−3.9 to −2.5)^††^	0.83 (0.80 to 0.86)^††^	−13.1 (−13.9 to −12.3)^††^	0.31 (0.28 to 0.34)^††^
**Sex**							
Male	16.5	14.7	5.2	−5.9 (−6.3 to −5.5)^††^	0.69 (0.67 to 0.71)^††^	−14.7 (−15.2 to −14.3)^††^	0.22 (0.20 to 0.24)^††^
Female	13.4	11.4	4.6	−4.6 (−5.0 to −4.3)^††^	0.68 (0.66 to 0.70)^††^	−10.9 (−11.3 to −10.4)^††^	0.26 (0.23 to 0.28)^††^
**Race and ethnicity**							
Hispanic or Latino	13.8	12.5	3.8	−6.9 (−7.7 to −6.1)^††^	0.64 (0.60 to 0.67)^††^	−15.8 (−16.7 to −14.9)^††^	0.18 (0.14 to 0.21)^††^
White, NH	15.6	13.5	5.0	−4.8 (−5.1 to −4.5)^††^	0.70 (0.69 to 0.72)^††^	−12.3 (−12.8 to −11.9)^††^	0.24 (0.22 to 0.26)^††^
Black or African American, NH	13.7	11.1	4.4	−5.6 (−6.2 to −5.0)^††^	0.65 (0.61 to 0.68)^††^	−11.7 (−12.6 to −10.8)^††^	0.26 (0.21 to 0.31)^††^
Asian, NH	14.8	14.6	5.4	−6.0 (−8.2 to −3.7)^††^	0.68 (0.59 to 0.78)^††^	−14.5 (−16.9 to −12.1)^††^	0.23 (0.15 to 0.31)^††^
Other, NH	16.4	15.1	6.5	−5.8 (−7.3 to −4.4)^††^	0.71 (0.65 to 0.78)^††^	−14.3 (−16.2 to −12.3)^††^	0.30 (0.21 to 0.38)^††^
Unknown	18.0	15.8	6.6	−7.8 (−10.1 to −5.4)^††^	0.67 (0.58 to 0.75)^††^	−17.2 (−20.0 to −14.4)^††^	0.26 (0.16 to 0.36)^††^
**No. of underlying medical conditions^§§^**							
0	2.8	3.1	0.6	0.7 (0.2 to 1.3)^††^	1.23 (1.06 to 1.41)^††^	−2.4 (−3.1 to −1.7)^††^	0.25 (0.05 to 0.45)^††^
1	8.1	7.8	2.9	−0.9 (−1.4 to −0.3)^††^	0.90 (0.84 to 0.96)^††^	−6.0 (−6.7 to −5.3)^††^	0.32 (0.25 to 0.39)^††^
2	15.4	12.0	4.3	−4.7 (−5.2 to −4.1)^††^	0.71 (0.68 to 0.74)^††^	−12.0 (−12.7 to −11.3)^††^	0.24 (0.21 to 0.28)^††^
3	22.3	15.2	5.3	−8.2 (−8.8 to −7.5)^††^	0.62 (0.60 to 0.65)^††^	−17.3 (−18.0 to −16.5)^††^	0.21 (0.18 to 0.23)^††^
4	25.6	16.8	6.2	−9.2 (−9.9 to −8.4)^††^	0.62 (0.59 to 0.64)^††^	−18.9 (−19.8 to −18.0)^††^	0.21 (0.18 to 0.24)^††^
≥5	26.0	16.8	6.6	−9.2 (−9.9 to −8.4)^††^	0.62 (0.60 to 0.65)^††^	−18.6 (−19.5 to −17.7)^††^	0.23 (0.20 to 0.26)^††^
**Disability^¶¶^**							
Yes	19.9	14.8	5.7	−5.0 (−5.6 to −4.4)^††^	0.70 (0.67 to 0.73)^††^	−12.4 (−13.1 to −11.6)^††^	0.26 (0.22 to 0.29)^††^
No	14.5	12.7	4.6	−5.3 (−5.6 to −5.1)^††^	0.68 (0.67 to 0.70)^††^	−13.0 (−13.3 to −12.6)^††^	0.23 (0.21 to 0.25)^††^

**Abbreviations:** ICD-10-CM = *International Classification of Diseases, Tenth Revision, Clinical Modification*; NH = non-Hispanic.

* Adjusted mortality risk differences and adjusted mortality risk ratios were estimated by a multivariable generalized estimating equation model specified as log-linked binomial with prediction errors adjusted for clustering at the hospital and patient level. The model included main effects and two-way interactions between pandemic period and the five variables in the table, plus insurance type, previous COVID-19, hospital U.S. Census Bureau region, and number of hospital beds.

^†^ Patients hospitalized primarily for COVID-19 had a primary discharge diagnosis of COVID-19 (i.e., ICD-10-CM code of U07.1) or a secondary discharge diagnosis of COVID-19 accompanied by either treatment with remdesivir or a primary discharge diagnosis of sepsis, pulmonary embolism, acute respiratory failure, or pneumonia.

^§^ Variant pandemic periods were selected based on two factors: 1) the U.S. epidemic curve for new admissions of patients with confirmed COVID-19 (https://covid.cdc.gov/covid-data-tracker/#new-hospital-admissions) and 2) the U.S. variant proportions from SARS-CoV-2 genomic surveillance (https://data.cdc.gov/Laboratory-Surveillance/SARS-CoV-2-Variant-Proportions/jr58-6ysp). Pandemic periods are defined using whole months because of date aggregation in the data source. The Delta variant (B.1.617.2) became the predominant circulating strain (representing >50% of sequenced isolates) during the week ending June 26, 2021, the Omicron B.1.1.529 subvariant became the predominant circulating strain during the week ending December 25, 2021, and the Omicron BA.2 subvariant became the predominant circulating strain during the week ending March 26, 2022. The predominant circulating strains during the early Omicron period were B.1.1.529 and BA.1 and during the later Omicron period were BA.2 and BA.2.12.1.

^¶^ August 2, 2022, data release. Data are from 678 hospitals that had at least one inpatient record per month during April 2020–May 2022.

** 95% CIs were calculated using SEs estimated via hospital-patient cluster bootstrap with 500 replications.

^††^ p<0.05.

**^§§^** Sixteen underlying medical conditions associated with higher risk for severe COVID-19 were assessed: asthma, cerebrovascular disease, cancer, chronic kidney disease, chronic lung disease, chronic liver disease, cystic fibrosis, dementia, diabetes, heart conditions, HIV, mental health disorder, obesity, primary immunodeficiencies, transplantation, and tuberculosis (https://www.cdc.gov/coronavirus/2019-ncov/science/science-briefs/underlying-evidence-table.html). Conditions were assessed using ICD-10-CM codes listed either at or before the COVID-19 health care encounter. For each patient, the number of underlying medical conditions was summed.

**^¶¶^** Presence of a disability was assessed using ICD-10-CM codes for birth defects, developmental disabilities, spinal cord injury, traumatic brain injury, and vision-, hearing-, and mobility-related disabilities.

## Discussion

During the period of Omicron variant predominance, the crude mortality risk among patients hospitalized primarily for COVID-19 decreased to 4.9% during April–June 2022, which is lower than any previous time in the pandemic and approximately one third of what it was during the period of Delta variant predominance^†††^ (*5*). In-hospital mortality decreased for all patient groups during the Omicron period and a larger proportion of hospitalizations and deaths occurred among populations most at risk for severe disease: patients aged ≥65 years and those with a disability or with three or more underlying medical conditions.^§§§^ Thus, in the later Omicron period, COVID-19 patients at lower risk were hospitalized less often and hospitalized COVID-19 patients at higher risk experienced less severe disease and lower mortality.

Several factors likely contributed to these favorable outcomes during the Omicron period, including higher levels of vaccine- and infection-induced immunity (*6*), advances in early treatment for patients at risk for severe disease,^¶¶¶^ and lower pathogenicity of Omicron subvariants (*7*). COVID-19 primary series and booster vaccination coverage was higher during the Omicron period than during the Delta period****; the effectiveness of receipt of 2 or 3 doses of COVID-19 mRNA vaccines against severe illness and death among hospitalized patients was 89% during the Delta period and 86% during the early Omicron period (*8*). In addition, the proportion of the U.S. population with infection-induced antibodies to SARS-CoV-2 increased from 33% in December 2021 to 57% by February 2022, indicating much higher infection-induced protection during the later Omicron period (*9*). Although oral COVID-19 antiviral therapies became available during the early Omicron period, their use increased substantially during the later Omicron period (*10*). These factors also likely contributed to reductions in other measures of disease severity observed during the later Omicron period, such as ICU admission and IMV.

Hospitalizations not primarily for COVID-19 were excluded from this study to allow for temporal comparison of mortality risk among persons hospitalized with COVID-19–related illness. The estimate derived from this study for hospitalizations not primarily for COVID-19 (37%) during January–March 2022 is within the range (12%–48%) reported by other sources derived from heterogeneous definitions and populations^††††^ (*5*). Of note, the observed difference in crude mortality risk between the early Omicron and Delta periods among hospitalizations primarily for COVID-19 was substantially less than the difference among total COVID-19 hospitalizations in this study and in a previous study (*2*). Thus, variation in the proportion of hospitalizations primarily for COVID-19 should be considered when interpreting past and future studies that compare hospitalization outcomes across pandemic periods.

The findings in this report are subject to at least five limitations. First, the definition of hospitalizations primarily for COVID-19 might be subject to misclassification, which could vary over time with changing patient and contextual factors. Second, COVID-19 vaccination status and previous COVID-19 are both under ascertained in PHD-SR; thus, the effect of SARS-CoV-2 immunity on mortality risk was not assessed. Third, disability status and number of underlying medical conditions might be misclassified because of reliance on ICD-10-CM codes. Fourth, PHD-SR data are incomplete for the later Omicron period; however, effect on mortality risk is expected to be minimal. Finally, although PHD-SR captures approximately 25% of annual U.S. hospital admissions, these findings might not be nationally generalizable.

In-hospital mortality risk was substantially lower during the later Omicron period overall and for older adults, persons with disabilities, and persons with multiple underlying medical conditions, who accounted for a larger proportion of hospitalizations in this period than they did during previous periods and remained at highest risk for death. It is uncertain whether patients with multiple underlying medical conditions are being hospitalized for respiratory complications from COVID-19 or for other acute or chronic conditions potentially exacerbated by SARS-CoV-2 infection. COVID-19–related hospitalizations and mortality should continue to be monitored as protective immunity evolves and new SARS-CoV-2 variants arise to inform public health guidance. Vaccination, early treatment, and appropriate nonpharmaceutical interventions remain important public health priorities to prevent severe COVID-19 illness and death, especially among persons most at risk (*1*).

**Summary**

**What is already known about this topic?**

Risk for severe COVID-19 increases with age, disability, and underlying medical conditions. The SARS-CoV-2 Omicron variant is more infectious but has been associated with less severe disease.

**What is added by this report?**

In-hospital mortality among patients hospitalized primarily for COVID-19 decreased from 15.1% (Delta period) to 4.9% (later Omicron period; April–June 2022), despite high-risk patient groups representing a larger proportion of hospitalizations. During the later Omicron period, the majority of in-hospital deaths occurred among adults aged ≥65 years (81.9%) and persons with three or more underlying medical conditions (73.4%).

**What are the implications for public health practice?**

Vaccination, early treatment, and appropriate nonpharmaceutical interventions remain important public health priorities to prevent COVID-19 deaths, especially among persons most at risk.

## Footnotes

* Examples include New York (https://www.wivb.com/news/new-york/new-york-state-covid-19-update-saturday-january-22/); Massachusetts (https://www.boston.com/news/coronavirus/2022/01/21/almost-half-of-mass-covid-hospitalizations-are-now-classified-as-incidental-heres-what-that-means/); Marin County, California (https://coronavirus.marinhhs.org/surveillance#keyindicators); and various health systems in Florida, Maryland, and Texas (https://www.nytimes.com/2022/01/04/health/covid-omicron-hospitalizations.html).

† Variant pandemic periods were selected based on two factors: 1) the U.S. epidemic curve for new admissions of patients with confirmed COVID-19 (https://covid.cdc.gov/covid-data-tracker/#new-hospital-admissions) and 2) the U.S. variant proportions from SARS-CoV-2 genomic surveillance (https://data.cdc.gov/Laboratory-Surveillance/SARS-CoV-2-Variant-Proportions/jr58-6ysp). Pandemic periods are defined using whole months because of date aggregation in the data source. Variants became the predominant circulating strain (representing >50% of sequenced isolates) during the following weeks: Delta (B.1.617.2) during the week ending June 26, 2021; Omicron B.1.1.529 during the week ending December 25, 2021; and Omicron BA.2 subvariant during the week ending March 26, 2022. Thus, the predominant circulating strains during the early Omicron period were B.1.1.529 and BA.1 and during the later Omicron period were BA.2 and BA.2.12.1.

§ PHD-SR is a large U.S. hospital-based all-payor database (http://offers.premierinc.com/rs/381-NBB-525/images/PHD_COVID-19_White_Paper.pdf), in which patient records are linked by a unique identifier within, but not across, hospital systems. This analysis included data from 678 hospitals that had at least one inpatient record per month during April 2020–May 2022. Of these, 521 hospitals also had at least one inpatient record during June 2021. PHD-SR data are released every 2 weeks; this study used the August 2, 2022, data release. According to information provided by Premier, Inc., data completeness is estimated to be 37%, 72%, 87%, and 95% during June, May, April, and January–March 2022, respectively.

¶ The definition of hospitalizations primarily for COVID-19 was intended to be relatively simple and replicable and used for monitoring temporal trends. Multiple iterations of the definition were evaluated, such as inclusion of additional primary discharge diagnoses and treatments. This definition was selected for its specificity in identifying patients experiencing COVID-19–related illness. The following ICD-10-CM codes were used to define sepsis (A41.89, A41.9, R65.2*), pneumonia (J12*, J18*), acute respiratory failure (J96.00, J96.01, J96.02, J96.20, J96.21, J96.22, J80, R06.03, R06.9, R09.2), and pulmonary embolism (I27.82, I26*).

** Sixteen underlying medical conditions associated with higher risk for severe COVID-19 were assessed: asthma, cerebrovascular disease, cancer, chronic kidney disease, chronic lung disease, chronic liver disease, cystic fibrosis, dementia, diabetes, heart conditions, HIV, mental health disorder, obesity, primary immunodeficiencies, transplantation, and tuberculosis (https://www.cdc.gov/coronavirus/2019-ncov/science/science-briefs/underlying-evidence-table.html). Conditions were assessed using ICD-10-CM codes listed either at or before the COVID-19 health care encounter. For each patient, the number of underlying medical conditions was summed and categorized. Presence of a disability was assessed separately from underlying medical conditions and included ICD-10-CM codes for birth defects, developmental disabilities, spinal cord injury, traumatic brain injury, and vision-, hearing-, and mobility-related disabilities. Previous COVID-19 was identified by presence of a COVID-19 diagnosis during an outpatient or inpatient encounter that occurred in the same hospital system ≥90 days before the current diagnosis. COVID-19 vaccination status was not assessed because it was underascertained in PHD-SR.

†† COVID-19 medications included dexamethasone, remdesivir, baricitinib, tofacitinib, tocilizumab, and sarilumab. Noninvasive ventilation included continuous positive airway pressure and bilevel positive airway pressure.

§§ The model included main effects and two-way interactions between pandemic period and the following covariates: age (0–17, 18–34, 35–49, 50–64, 65–79, and ≥80 years), sex (male and female), race and ethnicity (Hispanic or Latino [Hispanic], non-Hispanic White, non-Hispanic Black or African American [Black], non-Hispanic Asian, non-Hispanic other, and unknown), number of underlying medical conditions (0, 1, 2, 3, 4, and ≥5), and presence or absence of a disability. Additional covariates were included in the model without interaction terms: insurance type (commercial, Medicare, Medicaid, self-pay, and other or unknown), prevoius COVID-19, hospital U.S. Census Bureau region (Northeast, Midwest, South, and West), and number of hospital beds (<200, 200–499, and ≥500). Patients with unknown sex were excluded.

¶¶ From the regression, the following average predicted probabilities were obtained 1) the average predicted probability of death with pandemic period set to be later Omicron and all other covariates set to their observed values (PLO), 2) the average predicted probability of death with pandemic period set to be early Omicron and all other covariates set to their observed values (PEO), and 3) the average predicted probability of death with pandemic period set to be Delta and all other covariates set to their observed values (PD). aMRD is the difference in the average predicted probabilities (PLO minus PD and PEO minus PD). aMRR is the ratio of the average predicted probabilities (PLO divided by PD and PEO divided by PD). aMRRs and aMRDs were estimated for the full sample (where all covariates other than pandemic period were set at their observed values) and for each subsample (where the corresponding covariate was set at the specific category and other covariates were set at their observed values).

*** 45 C.F.R. part 46; 21 C.F.R. part 56; 42 U.S.C. Sect. 241(d); 5 U.S.C. Sect. 552a; 44 U.S.C. Sect. 3501 et seq.

†††
https://covid.cdc.gov/covid-data-tracker/#hospitalizations-severity (Accessed July 20, 2022).

§§§
https://www.cdc.gov/coronavirus/2019-ncov/hcp/clinical-care/underlyingconditions.html

¶¶¶
https://www.covid19treatmentguidelines.nih.gov/management/clinical-management-of-adults/nonhospitalized-adults--therapeutic-management/

**** https://covid.cdc.gov/covid-data-tracker/#vaccination-trends (Accessed July 20, 2022).

†††† https://www.governor.ny.gov/news/governor-hochul-updates-new-yorkers-states-progress-combating-covid-19-144; https://www.mass.gov/info-details/covid-19-response-reporting#covid-19-interactive-data-dashboard-
